# Targeting adipocytic discoidin domain receptor 2 impedes fat gain while increasing bone mass

**DOI:** 10.1038/s41418-021-00887-9

**Published:** 2021-10-13

**Authors:** Xiaoyu Yang, Jing Li, Liting Zhao, Yazhuo Chen, Zhijun Cui, Taotao Xu, Xu Li, Shufang Wu, Yan Zhang

**Affiliations:** 1 Center for Translational Medicine, The First Affiliated Hospital of Xi’an Jiaotong University, Xi’an, People’s Republic of China; 2 The Second Affiliated Hospital of Zhengzhou University, Zhengzhou, People’s Republic of China; 3 School of Basic Medical Sciences, Zhengzhou University, Zhengzhou, People’s Republic of China; 4 Department of Nutritional Sciences and Toxicology, University of California, Berkeley, Berkeley, CA USA; 5 The First Affiliated Hospital of Zhejiang Chinese Medical University, Hangzhou, People’s Republic of China

**Keywords:** Endocrine system and metabolic diseases, Drug development

## Abstract

Obesity is closely associated with low-bone-mass disorder. Discoidin domain receptor 2 (DDR2) plays essential roles in skeletal metabolism, and is probably involved in fat metabolism. To test the potential role of DDR2 in fat and fat-bone crosstalk, *Ddr2* conditional knockout mice (*Ddr2*^Adipo^) were generated in which *Ddr2* gene is exclusively deleted in adipocytes by Adipoq Cre. We found that *Ddr2*^Adipo^ mice are protected from fat gain on high-fat diet, with significantly decreased adipocyte size. *Ddr2*^Adipo^ mice exhibit significantly increased bone mass and mechanical properties, with enhanced osteoblastogenesis and osteoclastogenesis. Marrow adipocyte is diminished in the bone marrow of *Ddr2*^Adipo^ mice, due to activation of lipolysis. Fatty acid in the bone marrow was reduced in *Ddr2*^Adipo^ mice. RNA-Seq analysis identified adenylate cyclase 5 (Adcy5) as downstream molecule of Ddr2. Mechanically, adipocytic Ddr2 modulates Adcy5-cAMP-PKA signaling, and *Ddr2* deficiency stimulates lipolysis and supplies fatty acid for oxidation in osteoblasts, leading to the enhanced osteoblast differentiation and bone mass. Treatment of Adcy5 specific inhibitor abolishes the increased bone mass gain in *Ddr2*^Adipo^ mice. These observations establish, for the first time, that Ddr2 plays an essential role in the crosstalk between fat and bone. Targeting adipocytic Ddr2 may be a potential strategy for treating obesity and pathological bone loss simultaneously.

## Introduction

Obesity is one of the most important health problems facing modern society worldwide. More than 1.9 billion adults (18 years and older) are overweight, more than 650 million adults are obesity. This means that 39% of adults are overweight and 13% are obese. Obesity is closely associated with chronic diseases, such as high blood pressure, coronary heart disease, type 2 diabetes, and low-bone-mass disorders. Many obesity patients have osteoporosis symptom including decreased bone mass, changed microstructure, and increased bone fragility. Osteoporosis leads to increased risk of fracture and dramatically affects the life qualities of patients. Therefore, finding out a target that both regulates fat metabolism and bone mass is of great importance.

Bone marrow is an important component of the bone microenvironment. Bone marrow mesenchymal cells differentiate into osteoblast, adipocyte, or other lineages. The terminal differentiating cells communicate with each other. Adipocytes residing in the bone marrow (bone marrow adipocytes, BMAs), are indicated as paracrine and endocrine cells that contribute to local and systemic metabolism [1–3]. For example, Zou reported that marrow adipocytes regulate bone growth by altering bone morphogenetic protein (BMP) signaling [4]. BMAs communicate with bone cells locally, including osteoblasts, osteoclasts, and osteocytes, by secreting various factors [1]. Among these regulatory factors, lipids, and fatty acids are purported to be key players [5, 6].

Discoidin domain receptor 2 (Ddr2) is a receptor tyrosine kinase, which is activated by collagen binding [7]. Ddr2 is critical in skeletal homeostasis maintenance [8–11]. We and others demonstrated that Ddr2 promotes osteoblast differentiation and chondrocyte maturation via modulating runt related transcription factor 2 (Runx2) in an ERK MAPK-dependent manner [9–11]. Ddr2 is also implicated in fat metabolism [12–15]. *Ddr2* transgenic mice have decreased bodyweight, body mass index (BMI), and adipose amount [12]. Ddr2 controls differentiation direction of marrow progenitor cells to osteoblast or adipogenesis [13]. Evidence is accumulating that Ddr2 is involved in skeletal and fat physiology. However, the detailed mechanisms are not well understood.

In the present study, we found that knockout of *Ddr2* in adipocytes caused increase in bone mass. The lipolysis is enhanced and BMAs were diminished in bone marrow of *Ddr2*^Adipo^ mice. The adipocytic *Ddr2* ablation-induced enhancement of skeletal mass reflected Adcy5-cAMP-PKA-mediated lipolysis and fueling support for osteoblasts. These observations demonstrated that Ddr2 mediates the crosstalk between fat and bone. Targeting adipocytic Ddr2 may be a potential strategy for treating obesity and low-bone-mass disorders.

## Results

### Metabolic phenotype of *Ddr2*^Adipo^ mice

**Fig. 1:**
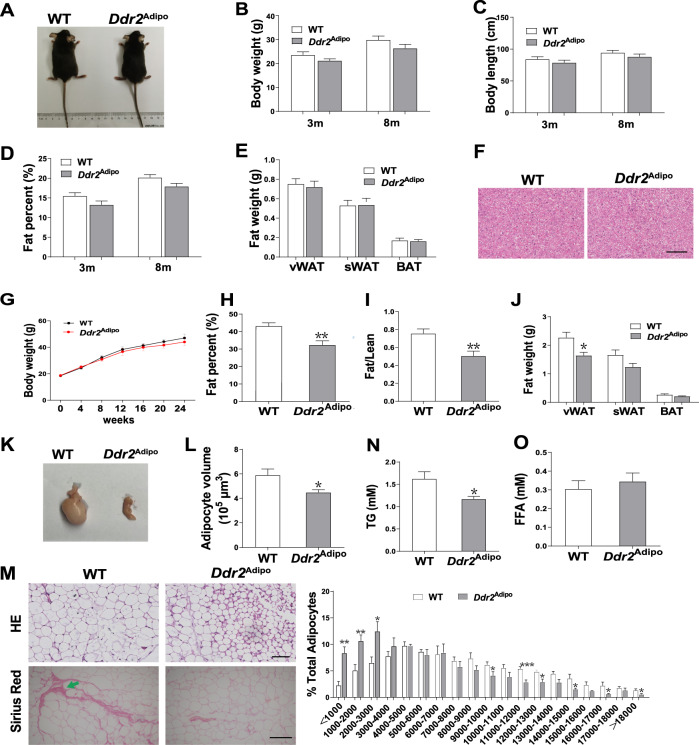
Metabolic phenotype of *Ddr2*^Adipo^ mice. **A** Appearance of 3-month-old *Ddr2*^Adipo^ and WT mice. **B** Bodyweight of *Ddr2*^Adipo^ and WT mice at the age of 3 and 8 month. **C** Body length of *Ddr2*^Adipo^ and WT mice. **D** Fat percent as determined by DEXA. **E** Weight of epididymal vWAT, inguinal sWAT, and BAT from *Ddr2*^Adipo^ and WT mice. **F** Representative H&E staining images of liver. Scale Bar = 100 μm**. G** Bodyweight of *Ddr2*^Adipo^ and WT mice during HFD feeding for 24 weeks. **H** Fat percent of *Ddr2*^Adipo^ and WT mice on HFD for 24 weeks. **I** Fat/lean ratio of *Ddr2*^Adipo^ and WT mice on HFD. **J** Weight of epididymal vWAT, inguinal sWAT, and BAT from *Ddr2*^Adipo^ and WT mice on HFD. **K** Appearance of epididymal vWAT from *Ddr2*^Adipo^ and WT mice on HFD. **L** Average volume of adipocytes in epididymal vWAT from *Ddr2*^Adipo^ and WT mice on HFD. **M** Representative H&E and Sirius Red staining images of epididymal vWAT. Arrow indicates the collagen accumulation. Adipocyte size distribution in epididymal vWAT. Scale Bar = 100 μm. **N** Serum TG of *Ddr2*^Adipo^ and WT mice on HFD. **O** Serum FFA of *Ddr2*^Adipo^ and WT mice on HFD. Data are presented as mean ± SEM. *n* = 6/group. *P < 0.05; **P < 0.01; ***P < 0.001 as determined by unpaired *t*-test.

Previous studies show that the body size and body length are increased while the bodyweight is decreased in *Ddr2* transgenic mice [12]. To find out the exact role of Ddr2 in fat metabolism, we generated *Ddr2* conditional knockout mice (*Ddr2*^Adipo^) in which *Ddr2* gene is exclusively deleted in adipocytes (Fig. S1). The effect of *Ddr2* deficiency on fat metabolism was investigated. Unexpectedly, in contrast to the phenotypes of globally *Ddr2* modified mice, there is no difference in body size, weight, and length between *Ddr2*^Adipo^ mice and wild-type (WT) controls, at age of 3 and 8 month (Fig. 1A–C). The body fat rate as determined by dual energy X-ray absorptiometry (DEXA) remains normal in *Ddr2*^Adipo^ mice (Fig. 1D). The weight of fat is also similar in *Ddr2*^Adipo^ mice compared with controls (Fig. 1E). No obvious changes were found in livers of *Ddr2*^Adipo^ mice (Fig. 1F). Then WT and *Ddr2*^Adipo^ mice were fed with high-fat diet (HFD) for 24 weeks. The gain of body weights induced by HFD is similar in *Ddr2*^Adipo^ mice, with similar food intake (Fig. 1G and Fig. S2). However, the body fat rate is significantly decreased as determined by DEXA (Fig. 1H). Consistently, the fat/lean ratio is significantly reduced in *Ddr2*^Adipo^ mice fed an HFD (Fig. 1I). The weight of visceral fat in *Ddr2*^Adipo^ mice is significantly reduced (Fig. 1J), as well as the volume of adipocytes (Fig. 1K and Fig. 1L). *Ddr2*^Adipo^ visceral adipocytes are much smaller and have less collagen accumulation. The adipocyte size distribution in *Ddr2*^Adipo^ mice was shifted to more smaller ones (Fig. 1M). Serum triglyceride (TG) level is decreased (Fig. 1N) whereas free fatty acid (FFA) remained unchanged (Fig. 1O) in the HFD fed *Ddr2*^Adipo^ mice. Collectively, these results demonstrated that *Ddr2*^Adipo^ mice were protected from HFD-induced fat gain.

### *Ddr2*^Adipo^ mice have increased bone mass and bone strength

**Fig. 2:**
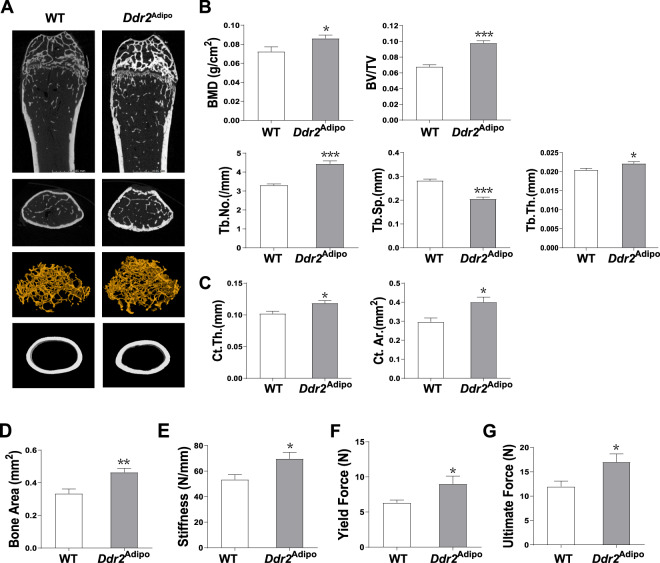
*Ddr2*^Adipo^ mice have increased bone mass. **A** μCT images of femurs of 3-month-old *Ddr2*^Adipo^ and WT littermates. **B** Quantitative μCT analysis of trabecular bone. BMD bone mineral density, BV/TV bone volume per tissue volume, Tb.No, trabecular number, Tb.Sp. trabecular separation, Tb.Th. trabecular thickness. **C** Quantitative μCT analysis of cortical bone. Ct. Th. cortical thickness, Ct. Ar. cortical area. **D** μCT analysis of diaphyseal bone area. **E** Bending tests analysis of *Ddr2*^Adipo^ and WT femoral stiffness. **F** Yield Force of *Ddr2*^Adipo^ and WT femoral bone. **G** Ultimate force. Data are presented as mean ± SEM. *n* = 6 femurs from 6 mice for WT mice, *n* = 7 femurs from 7 mice for *Ddr2*^Adipo^ mice. *P < 0.05; **P < 0.01; ***P < 0.001 as determined by unpaired *t*-test.

It is reported that slie mice which contain nonfunctional *Ddr2* allele have multiple skeletal defects [13, 16]. To find out whether adipose-specific deletion of *Ddr2* has similar effects on bone, femurs were collected for analysis at the age of 3 months. As shown in μCT images, the bone mass of *Ddr2*^Adipo^ mice is significantly increased compared to their WT controls (Fig. 2A). The parameters including bone mineral density (BMD), bone volume/total volume (BV/TV) ratio, trabecular number (Tb. No.), and trabecular thickness (Tb. Th.) are significantly augmented (Fig. 2B). Femoral cortical thickness (Ct. Th.) and cortical area (Ct. Ar.) of *Ddr2*^Adipo^ mice are increased as well (Fig. 2C). Increased bone mass usually amplifies the mechanical properties at the whole-bone level. Therefore, three-point bending tests were performed to determine the bone strength. In consistent with increased bone area (Fig. 2D), femurs of 12-weeks-old *Ddr2*^Adipo^ mice are significantly stiffer (Fig. 2E) with enhanced yield force (Fig. 2F). The ultimate force of *Ddr2*^Adipo^ femurs are significantly increased (Fig. 2G), indicating the enhanced whole-bone strength. Collectively, these data showed that bone mass and mechanical properties are significantly induced in *Ddr2*^Adipo^ mice.

### Bone formation is stimulated in *Ddr2*^Adipo^ mice

**Fig. 3:**
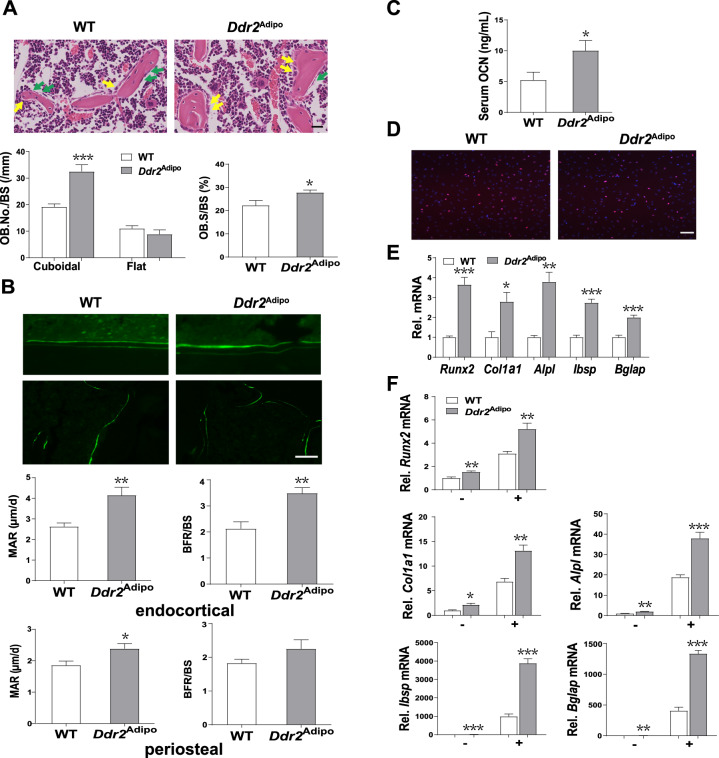
Bone formation is stimulated in *Ddr2*^Adipo^ mice. **A** Representative H&E staining images of femur trabecular bones of 3-month-old *Ddr2*^Adipo^ and WT littermates. Yellow and green arrows indicate cuboidal and flat osteoblasts, respectively. Quantification of osteoblast number per bone surface (OB. No./BS) and osteoblast surface per bone surface (OB. S/BS). *n* = 6 femurs from 6 mice for each group. Scale bar = 30 μm. **B** Fluorescence microscopic images of distal femur of *Ddr2*^Adipo^ and WT mice administered time-spaced calcein. Histomorphometric analysis of mineral apposition rate (MAR) and bone formation rate per bone surface (BFR/BS) of trabecular (up) and cortical (bottom) bone. *n* = 6 femurs from 6 mice for each group. Scale bar = 30 μm. **C** Serum OCN level of *Ddr2*^Adipo^ and WT mice. *n* = 6/group. **D** EdU incorporation in the calvaria cells of *Ddr2*^Adipo^ and WT mice. *n* = 6/group. Scale bar = 100 μm. **E** mRNA expression of osteoblast differentiation markers in femurs of 4-week-old *Ddr2*^Adipo^ and WT mice. *n* = 6/group. **F** mRNA expression of osteoblast differentiation markers in calvaria cells of *Ddr2*^Adipo^ and WT mice, with or without 14-day induction. *n* = 6/group. Data are presented as mean ± SEM. *P < 0.05; **P < 0.01; ***P < 0.001 as determined by unpaired *t*-test.

To investigate whether the augmented bone mass was due to enhanced osteogenesis, decreased resorption, or both, we detected the effects of *Ddr2* deficiency on bone formation firstly. Hematoxylin and eosin (H&E) staining images of long bones showed that there are more cuboidal osteoblasts, which are considered to actively synthesize bone, in *Ddr2*^Adipo^ mice than WT controls (Fig. 3A). Time-spaced course calcin was administrated and histomorphometric analysis was conducted. Data showed that trabecular bone formation is accelerated in *Ddr2*^Adipo^ mice as manifest by activity of individual osteoblasts (mineral apposition rate, MAR) as well when expressed in the context of trabecular surface (bone formation rate/bone surface, BFR/BS) (Fig. 3B).

Consistently, serum osteocalcin is markedly increased in the *Ddr2*^Adipo^ mice (Fig. 3C). 5-bromodeoxyuridine (BrdU) incorporation assay showed that the osteoblast proliferation is not affected after *Ddr2* depletion, excluding the possibility that stimulated bone formation is due to increased osteoblast number (Fig. 3D). Additionally, mRNAs levels of osteoblastic markers including *Runx2*, collagen1 α1 (*Col1α1*), alkaline phosphatase (*Alpl*), integrin binding sialoprotein (*Ibsp*), and bone gamma carboxyglutamate protein (*Bglap*) in bone tissues (Fig. 3E) and primary calvaria cells (Fig. 3F), are significantly increased in *Ddr2*^Adipo^ mice. This results further suggest that stimulated osteoblast activity contributes to the osteosclerotic phenotype of *Ddr2*^Adipo^ mice.

### Bone resorption is promoted in *Ddr2*^Adipo^ mice

**Fig. 4:**
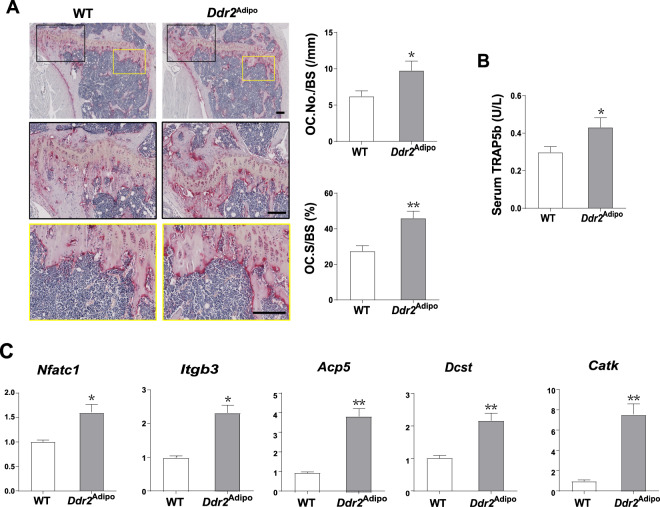
Bone resorption is promoted in *Ddr2*^Adipo^ mice. **A** TRAP staining of femurs of 3-month-old *Ddr2*^Adipo^ and WT littermates. Histomorphometric analysis of osteoclast number per bone surface (OC. No./BS) and osteoclast surface per bone surface (OC. S/BS) of *Ddr2*^Adipo^ and WT femurs. *n* = 5 femurs from 5 mice for each group. Scale Bar = 100 μm. **B** Serum TRAP5b of *Ddr2*^Adipo^ and WT mice. *n* = 6/group. **C** mRNA expression of osteoclast differentiation markers in femurs of 4-week-old *Ddr2*^Adipo^ and WT mice. *n* = 6/group. Data are presented as mean ± SEM. *P < 0.05; **P < 0.01 as determined by unpaired *t*-test.

The increased bone mass of *Ddr2*^Adipo^ mice may be caused by both enhanced bone formation and decreased bone resorption. Moreover, bone resorption is also under the regulation of bone formation, which is called coupling regulation. Thus, we detected the effects of *Ddr2* deficiency on bone resorption. Tartrate-resistant acid phosphatase (TRAP) staining results indicated the increased osteoclast abundance in *Ddr2*^Adipo^ mice compared to their WT counterparts (Fig. 4A). The increased number (OC. No./BS) and surface (OC. S./BS) of osteoclasts per bone surface is further confirmed by histomorphometry analysis (Fig. 4A). As expected, circulating tartrate-resistant acid phosphatase 5b (TRAP5b), a classical marker of osteoclastogenesis, is also significantly elevated in *Ddr2*^Adipo^ mice (Fig. 4B). mRNA levels of osteoclastic markers, including nuclear factor of activated T cells, cytoplasmic, calcineurin-dependent 1 (*Nfatc1*), integrin beta 3 (*Itgb3*), acid phosphatase 5, tartrate resistant (*Acp5*), DC-STAMP domain containing 1 (*Dcst1*), and cathepsin K (*Ctsk*) are significantly upregulated in *Ddr2*^Adipo^ bones (Fig. 4C). These results demonstrated that osteoclastic bone resorption is also stimulated in *Ddr2*^Adipo^ mice. Given the net gain of bone mass in *Ddr2*^Adipo^ mice, we concluded that the positive effect of stimulated bone formation is surpassing the negative effect of stimulated resorption. Collectively, *Ddr2*^Adipo^ mice have increased bone mass due to the great acceleration of bone formation.

### Bone marrow fat is diminished in *Ddr2*^Adipo^ mice

To find out the underlying mechanism of stimulated osteoblast differentiation and increased bone mass in *Ddr2*^Adipo^ mice, we examined the level of serum adipokines to see whether the systematic metabolic changes leads to the skeletal phenotype. The serum level of adiponectin (Fig. S3A), leptin (Fig. S3B), and insulin (Fig. S3C) remains normal in *Ddr2*^Adipo^ mice. Glucose tolerance, indicating the ability of clearing glucose following insulin challenge, remains unchanged in *Ddr2*^Adipo^ mice (Fig. S3D). Insulin tolerance, indicating the ability of clearance of glucose following insulin challenge, also remains normal (Fig. S3E). Consisting with the reduced body fat mass in *Ddr2*^Adipo^ mice on HFD, *Ddr2*^Adipo^ mice are protected from HFD-induced glucose intolerance (Fig. S3F), and exhibit enhanced clearance of glucose following insulin challenge (Fig. S3G). These results indicate that osteosclerosis of *Ddr2*^Adipo^ mice is not caused by the metabolic syndrome.

Previous study indicates that global *Ddr2* deficiency regulates the differentiation fate of bone marrow stromal cells (BMSCs) [13], then we detect whether adipocytic deletion of *Ddr2* affects the differentiation of BMSCs into osteoblasts or adipocytes. As shown in Fig. S4A, the mRNA levels of osteoblastic markers remain unchanged in *Ddr2*^Adipo^ BMSCs treated with osteoblastic differentiation inducers. The expression of adipocytic markers are also similar between *Ddr2*^Adipo^ and WT cells (Fig. S4B). These results demonstrated that adipocytic *Ddr2* deficiency does not have any feedback effects on BMSC differentiation, and stimulated osteoblast differentiation in *Ddr2*^Adipo^ mice is not caused by lineage selection of BMSCs.

**Fig. 5:**
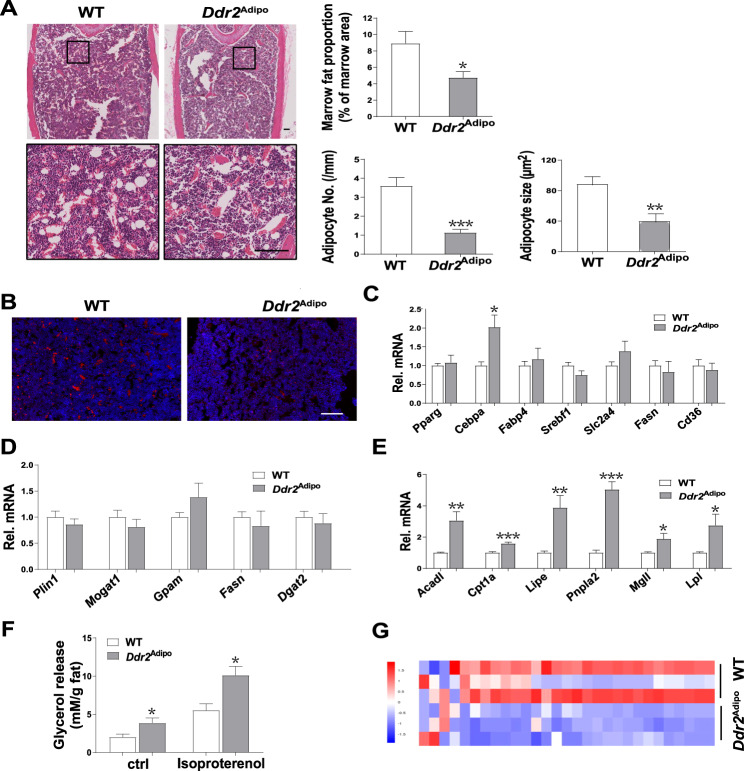
Bone marrow fat is diminished in *Ddr2*^Adipo^ mice. **A** Representative H&E staining images of femur trabecular bones of 8-month-old *Ddr2*^Adipo^ and WT littermates. Marrow fat proportion, adipocyte number and size as determined by Bioquant software. *n* = 6 femurs from 6 mice for each group. Scale Bar = 100 μm. **B** Immunofluorescence staining of Perilipin 1 in femur sections of 8-month-old *Ddr2*^Adipo^ and WT mice. **C** mRNA expression of lipogenic genes in bone marrow adipocytes. *n* = 6/group. **D** mRNA expression of lipid storage genes. *n* = 6/group. **E** mRNA expression of lipolytic genes. *n* = 6/group. **F** Glycerol released from bone marrow adipocytes treated with DMSO or isoproterenol. *n* = 6/group. **G** Heatmap of hierarchical clustering analysis of fatty acid in bone marrow of *Ddr2*^Adipo^ and WT mice. Data are presented as mean ± SEM. *P < 0.05; **P < 0.01; ***P < 0.001 as determined by unpaired *t*-test.

Bone marrow adipocytes is one of the essential components in the local microenvironment for osteoblast differentiation. Histological analysis shows that there is substantially less marrow adipose tissue in femurs of *Ddr2*^Adipo^ mice (Fig. 5A). Marrow fat proportion, adipocyte number, and adipocyte size are significantly decreased in *Ddr2*^Adipo^ mice (Fig. 5A). Consistently, Perilipin 1 (Plin1) staining positive cells, which indicates the adipocyte in the bone marrow are obviously decreased in *Ddr2*^Adipo^ mice (Fig. 5B). Bone marrow adipocytes were isolated via negative and subsequent positive selection using a commercial kit, and mRNA expression was determined. Lipogenic genes levels are similar between *Ddr2*^Adipo^ and WT cells, except CCAAT/enhancer binding protein alpha (*Cebpa*, Fig. 5C). Lipid storage genes, including *Plin1*, monoacylglycerol O-acyltransferase 1 (*Mogat1*), glycerol-3-phosphate acyltransferase, mitochondrial (*Gpam*), fatty acid synthase (*Fasn*), diacylglycerol O-acyltransferase 2 (*Dgat2*) remain unchanged (Fig. 5D). Lipolytic genes, including acyl-Coenzyme A dehydrogenase, long-chain (*Acadl*), carnitine palmitoyltransferase 1a (*Cpt1a*), lipase, hormone sensitive, (*Lipe*), patatin-like phospholipase domain containing 2 (*Pnpla2*), monoglyceride lipase (*Mgll*), and lipoprotein lipase (*Lpl*) increases significantly (Fig. 5E). *Ddr2* deletion stimulates glycerol mobilization in the absence and presence of isoproterenol, indicating the enhanced lipolysis (Fig. 5F). The FFA abundance in the bone marrow were detected. Various kinds of long-chain fatty acids diminished in the bone marrow of *Ddr2*^Adipo^ mice (Fig. 5G).

### Adcy5 mediated lipolysis is activated in *Ddr2*^Adipo^ mice

**Fig. 6:**
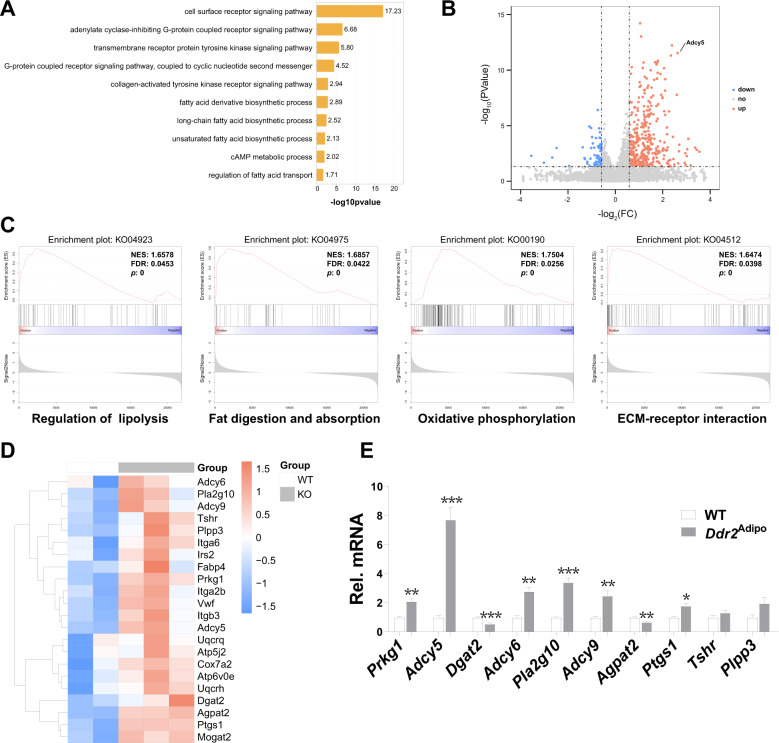
Lipolysis is activated in *Ddr2*^Adipo^ mice. **A** Gene Ontology (GO) term analysis of all differentially expressed genes based on RNA-seq data. **B** Volcano plot analysis of all differentially expressed genes. “*Adcy5*” was labeled. **C** Geneset enrichment analysis (GSEA). **D** Heatmap of differentially expressed genes based on GSEA with adjusted *P* < 0.05. **E** Validation of differentially expressed genes by qPCR. Data are presented as mean ± SEM. *n* = 6/group. *P < 0.05; **P < 0.01; ***P < 0.001 as determined by unpaired *t*-test.

To investigate the underlying mechanism, we characterized the transcriptomic profiles of BMAs by performing unbiased RNA sequencing (RNA-seq). Gene Ontology (GO) term analysis of all differentially expressed genes was performed based on RNA-seq data. Data revealed that differential expressed gene were associated with cell surface receptor signaling pathway, adenylated cyclase-inhibiting G protein-coupled receptor signaling pathway, and G protein-coupled receptor signaling pathway, coupled to cyclic nucleotide second messenger (Fig. 6A). Volcano plot analysis of all differentially expressed genes revealed that most differentiate expressed genes (|log_2_Fold Change | > 0.584963, −log_10_ *P*-value > 1.3) were upregulated in *Ddr2*^Adipo^ mice (Fig. 6B). Gene Set Enrichment Analysis confirmed significant enrichment of dysregulated genes involved in regulation of lipolysis, fat digestion and absorption, oxidative phosphorylation, and ECM-receptor interaction (Fig. 6C). Further analysis identified the 24 most upregulated and downregulated genes involved in these processes in a heatmap (Fig. 6D), and qPCR confirmed these genes and indicated that Adcy5 was the most significantly upregulated one (Fig. 6E). Adcy5 was also labeled in volcano plot analysis (Fig. 6B). In conclusion, these data suggested lipolysis and Adcy5 may be the causes of the stimulated lipolysis in *Ddr2*^Adipo^ mice.

### Adipocytic Ddr2 modulates bone mass through Adcy5-cAMP-PKA pathway

**Fig. 7:**
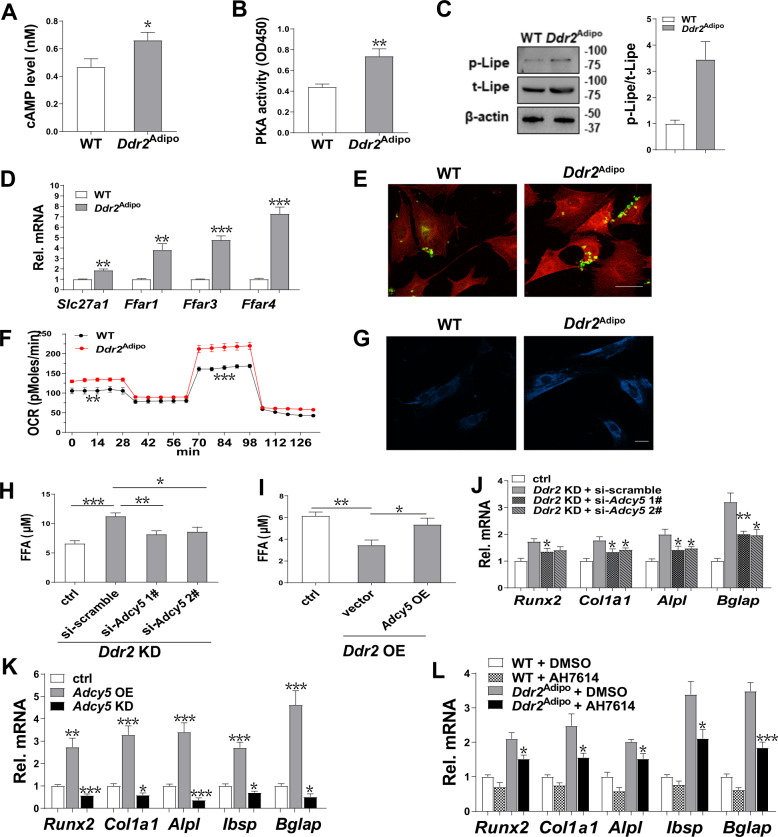
Adipocytic Ddr2 modulates bone mass though Adcy5-cAMP-PKA pathway. **A** cAMP levels in bone marrow adipocytes of *Ddr2*^Adipo^ and WT littermates. *n* = 6/group. **B** PKA activity in bone marrow adipocytes. *n* = 6/group. **C** Phosphorylated and total Lipe level determined by western blotting. **D** mRNA expression of fatty acid transporter genes in calvaria cells. *n* = 6/group. **E** Fatty acid uptake by calvaria cells. Co-culture 3T3-L1 cells with cherry-labeled WT or *Ddr2*^Adipo^ calvaria cells, and FFA was stained with BODIPY. Scale bar = 25 μm. **F** Oxygen consumption rate (OCR) in calvaria cells was determined by XF Cell Mito Stress Assay. *n* = 12/group. **G** Activity of fatty acid oxidation in calvaria cells determined by FAOBlue staining. Scale bar = 25 μm. **H** *Adcy5* knockdown inhibits FFA release induced by *Ddr2* depletion. *n* = 6/group. **I** *Adcy5* overexpression rescues *Ddr2* inhibition of FFA release. *n* = 6/group. **J** *Adcy5* knockdown blocks the stimulated expression of osteoblastic genes induced by *Ddr2* deficiency. *n* = 6/group. **K** Adipocytic Adcy5 modulates osteoblastic differentiation. *n* = 6/group. **L** Inhibition of FFA receptor blocks the *Ddr2* depletion induced osteoblast differentiation. The culture media of differentiated 3T3-L1 cells was incubated with calvaria cells in the absence or presence of FFA receptor inhibitor AH7614. *n* = 6/group. Data are presented as mean ± SEM. *P < 0.05; **P < 0.01; ***P < 0.001 as determined by unpaired *t*-test.

To investigate the detailed mechanism, we assayed cAMP level of bone marrow adipocytes, given cAMP is an important downstream effector of Adcy5 [17]. Data revealed that cAMP level was significant elevated in *Ddr2*^Adipo^ mice (Fig. 7A). PKA is a cAMP-dependent kinase that could activate Lipe. Then we determined PKA activity and Lipe phosphorylation in bone marrow adipocytes. PKA activity (Fig. 7B) and Lipe phosphorylation are significantly induced in bone marrow adipocytes of *Ddr2*^Adipo^ mice (Fig. 7C). Similar regulations were also observed in 3T3-L1 cells (Fig. S5A–E). Consistently, the FFA concentration in the culture media of *Ddr2* knockdown 3T3-L1 cells was significantly increased (Fig. S5F).

mRNA expression of fatty acid transporter genes including solute carrier family 27 (fatty acid transporter), member 1 (*Slc27a1*), free fatty acid receptor 1 (*Ffar1*), free fatty acid receptor 3 (*Ffar3*), and free fatty acid receptor 4 (*Ffar4*) are significantly elevated in calvaria cells of *Ddr2*^Adipo^ mice (Fig. 7D). Calvaria cells were labeled by overexpressing Cherry, and cocultured with 3T3-L1 cells. As shown in Fig. 7E, FFA uptake by *Ddr2*^Adipo^ calvaria cells, as determined by BODIPY staining, was significantly induced. Fatty acid was reported to stimulate mitochondrial oxidative phosphorylation in osteoblasts [18–20]. Then we detected the oxygen consumption rate (OCR) in calvaria cells by XF Cell Mito Stress assay. As shown in Fig. 7F, *Ddr2* deficiency in adipocytes caused basal and maximal respiration greatly induced in calvaria cells of *Ddr2*^Adipo^ mice. Fatty acids oxidation activity, as determined by FAOBlue staining, is obviously enhanced in *Ddr2*^Adipo^ calvaria cells (Fig. 7G).

To further investigate the involvement of Adcy5 in Ddr2 regulation of lipolysis, *Adcy5* expression was interfered in *Ddr2*-depleted 3T3-L1 cells. *Adcy5* knockdown significantly downregulates FFA release in *Ddr2*-depleted 3T3-L1 cells (Fig. 7H). On the contrary, Overexpression of *Adcy5* restores the FFA release in *Ddr2*-overexpressing cells (Fig. 7I). In the co-culture assay of 3T3-L1 cells and calvaria cells, *Adcy5* knockdown in *Ddr2*-depleted 3T3-L1 cells significantly rescues the induced expression of osteoblastic maker genes in *Ddr2*^Adipo^ calvaria cells (Fig. 7J). Furthermore, being cocultured with *Adcy5* overexpression 3T3-L1 cells, the osteoblastic gene expression in calvaria cells was significantly stimulated, while *Adcy5 knockdown* caused significant downregulation (Fig. 7K).

Ffar4 was identified as the most significantly increased fatty acid receptor (Fig. 7D), then we used Ffar4 inhibitor AH7614 to blocked FFA uptake in calvaria cells. *Ddr2*^Adipo^ calvaria cells have increased expression of osteoblastic genes compared to their WT counterparts (Fig. 7L, 3F). However, in the presence of AH7614, the upregulation of osteoblastic expression gene in *Ddr2*^Adipo^ calvaria cells was significantly inhibited (Fig. 7L).

**Fig. 8:**
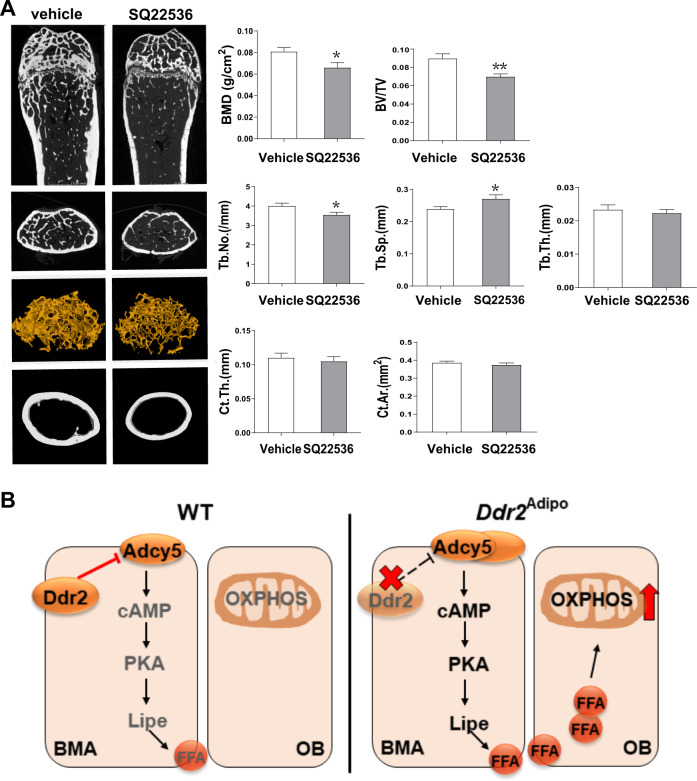
Adenylyl cyclase inhibition blocks *Ddr2*^Adipo^ bone phenotypes. **A** Quantitative μCT analysis of femurs of *Ddr2*^Adipo^ mice treated with or without Adenylyl cyclase inhibitor SQ22536. *n* = 6 femurs from 6 mice for each group. Data are presented as mean ± SEM. *P < 0.05; **P < 0.01 as determined by unpaired *t*-test. **B** Schematic overview of bone-fat crosstalk mediated by Ddr2. In bone marrow microenvironment, bone marrow adipocytes modulate osteoblasts. In WT mice, the expression level of Adcy5 is low, due to inhibition by Ddr2. The cAMP-PKA signaling remains in an inactive status. In *Ddr2*^Adipo^ mice, expression of Adcy5 is increased, leading to stimulated cAMP-PKA pathway and subsequent increased lipolysis and fatty acid release. Uptake of fatty acid by nearby osteoblasts stimulates the mitochondrial OXPHOS and provides more energy for osteoblast differentiation and bone formation. BMA bone marrow adipocyte, OB osteoblast, cAMP cyclic adenosine monophosphate, PKA protein kinase A.

Then we used an adenylyl cyclase inhibitor SQ22536, to test the effects of Adcy5 and cAMP inhibition on bone mass of *Ddr2*^Adipo^ mice in vivo. As shown in μCT images, after 2-month treatment of SQ22536, the bone mass of *Ddr2*^Adipo^ mice is significantly decreased compared to vehicle controls (Fig. 8A). BMD, BV/TV, and Tb. No. are significantly dropped, whereas Tb. Sp. are significantly elevated (Fig. 8A). However, femoral Ct. Th. and Ct. Ar. remain unchanged (Fig. 8A). Taken together, our data demonstrated that Adcy5 mediates the Ddr2 regulation of adipocyte lipolysis, and Ddr2 deficiency leads to Adcy5 upregulation and consequent lipolysis and FFA release, which in turn stimulates osteoblast differentiation and bone mass gain.

## Discussion

Obesity and osteoporosis are both common conditions with high rates of morbidity and mortality [21]. The relationship between obesity and bone is complex [22, 23]. Obesity protects against fractures in some sites while raising the risk in other parts of the body [22–24]. Obesity people with metabolic syndrome often have osteoporosis related problems [21]. Adipose tissue, especially visceral fat, negatively associated with bone mass [4, 23, 25]. This prompt us to investigate the essential player that mediates the crosstalk between fat and bone, aiming to disclose novel anti-obesity and anti-osteoporosis therapeutic strategies.

In this study, we showed that *Ddr2* ablation in adipocytes induced an increase in bone mass and mechanical properties. Bone formation and resorption were stimulated, while lipolysis was induced in *Ddr2*^Adipo^ mice. Mechanistically, Ddr2 regulates Adcy5 and downstream cAMP-PKA signaling in adipocytes, and indirectly affects osteoblast differentiation by controlling the release of fatty acid from lipolysis and regulation of fatty acid oxidation in osteoblasts. As the working model shown in Fig. 8B, in WT mice, the expression level of Adcy5 is low, due to inhibition by Ddr2. The cAMP-PKA signaling remains in an inactive status. In *Ddr2*^Adipo^ mice, expression of Adcy5 is increased, leading to stimulated cAMP-PKA pathway and subsequent increased lipolysis and fatty acid release. Uptake of fatty acid by nearby osteoblasts stimulates the mitochondrial oxidative phosphorylation (OXPHOS) and provides more energy for osteoblast differentiation and bone formation. Collectively, our results reveal a novel role of Ddr2 in fat-bone crosstalk, and providing a potential therapeutic strategy for obesity and osteoporosis.

Different from the phenotype of global knockout or slie mice [16, 26, 27], *Ddr2*^Adipo^ mice show increased bone mass. *Ddr2* is widely expressed in mesenchymal cells [7]. Therefore, it is probably that adipocytic Ddr2-mediated bone mass suppression is counteracted by stimulation effects of Ddr2 in other tissues/cells in globally knockout mice. The net effects of *Ddr2* deficiency is decline in bone mass. Bone formation is stimulated in *Ddr2*^Adipo^ mice, attributing to enhanced osteoblast differentiation but not proliferation. Osteoblasts provide necessary signal for osteoclast function [28]. Receptor activator of nuclear factor-κ B ligand (RNAKL) on the cellular membrane of osteoblasts is indispensable for the differentiation of osteoclasts [29, 30]. Therefore, the activated bone resorption we observed may be an accompanying phenomenon after the enhanced bone formation.

On chow diet, the bodyweight and glucose tolerance of *Ddr2*^Adipo^ and WT mice are comparable. Although HFD-induced weight gain is also similar, *Ddr2*^Adipo^ mice have lower body fat rate and better sensitivity to glucose and insulin challenge on HFD. The difference in fat metabolism on chow diet need further investigation using appropriate experimental means, despite decreased bone marrow fat was detected in the old age *Ddr2*^Adipo^ mice. Zou and colleagues reported that fat-free mice, which completely lack fat, have systemic increased bone mass [25]. Recently, Zou also reported that postnatal adipocyte elimination yields profound increase in bone mass, due to absence of BMAs [4]. These results are consistent with ours, which indicated that BMAs provide energy for osteoblast differentiation in the bone marrow microenvironment.

BMA content inversely correlates with bone mass [31, 32]. BMAs and osteoblasts are derived from the same progenitors, mesenchymal cells. Signals promoting adipogenesis impair osteogenesis, while signals inhibiting adipogenesis promote differentiation of osteoblasts [33, 34]. Additionally, BMAs secrete factors that regulate osteogenesis [2]. In vitro evidence demonstrated that co-culture of osteoblast progenitors with either primary adipocytes or differentiated 3T3-L1 adipocytes decreases the expression of osteoblastic makers [35]. In vivo, adipogenic cells also inhibit bone cells by secreting factors, including adipokines, extracellular vesicles, pro-inflammatory cytokines, and fatty acids [1, 2]. However, the detailed mechanism underlying which molecule drives the production of these factors, especially fatty acids, remains to be clarified. Here, we revealed the involvement of Ddr2, further supporting the direct regulation of bone cells by BMAs.

Increasing evidence implicates Ddr2 in fat metabolism. Activating mutation of *DDR2* causes Warburg-Cinotti Syndrome, in which wasting subcutaneous tissue and acro-osteolysis are observed [14]. A functional crosstalk between Ddr2 and insulin/insulin-like growth factor system (IIGFs) was discovered recently [36, 37]. Slie mutant mice show a robust reduction of Igf1 expression in the liver [16]. Ddr2 modulates insulin responsiveness in preadipocytes and adipocytes. Overexpressing *Ddr2* in 3T3-L1 adipocytes leads to reduction of insulin-stimulated IRS-1 tyrosine phosphorylation and glucose transporter, thereby insulin resistance [15]. On the other side, insulin also promotes Ddr2 phosphorylation [36, 37]. After Igf2 exposure, Ddr2 is recruited to the IR-A and reaches maximal activation [36, 37]. As an important insulin target organ/tissue, adipose tissue requires insulin signaling to be fully functional [38, 39]. It is reasonable that Ddr2 employs Adcy5 to participate in the crosstalk with insulin signaling in adipocytes. Further investigation is needed to clarify the detailed mechanism.

Adcy5 belongs to the adenylyl cyclase families (ACs), which catalyze the conversion of ATP into the second messenger cAMP [17, 40]. ACs partner with G protein-coupled receptors (GPCRs) and downstream effectors, creating discrete signaling complexes [17, 40]. Adcy5 plays essential roles in heart and brain [41]. Adcy5 associates with caveolar and lipid raft microdomains [42]. *Adcy*5^−/−^ mice exhibit decreased bodyweight on standard and high-fat diet, with improved glucose tolerance and increased insulin sensitivity [43, 44]. Numerous studies linked *Adcy5* gene polymorphisms to altered glucose metabolism, diabetes, and obesity [45, 46]. Adcy5 is activated by the G-protein Gαs, PKC, and Raf kinase, while be inhibited by Gαi and Ca^2+^ [17, 40]. In this study, we demonstrated that Ddr2 regulates Adcy5 and downstream signaling in adipocytes. As far as we know, until now, there is no report regarding the regulation of Adcy5 by Ddr2. Our results uncover a novel regulation mechanism for Adcy5. We previously indicated that Ddr2 interacts with Nrp1 on the cellular membrane, leading to mutual regulation of each other in osteoclasts [8]. In osteoblasts, Ddr2 regulates Runx2 phosphorylation via ERK MAPK [9]. These two kinds of Ddr2-mediated regulation are totally different. The former one is mediated by protein-protein binding, while the latter one is depending on Ddr2’s tyrosine kinase activity. Using RNA-Seq techniques, we identified Adcy5 as Ddr2’ downstream molecule. We also successfully used its inhibitor SQ22536 to rescue the bone phenotype caused by *Ddr2* deficiency, further supporting the Ddr2-Adcy5 regulation. However, whether this regulation is direct or indirect, and whether it depends on Ddr2’s kinase activity or not, remains to be further investigated. Our future study will focus on this point and investigate the detailed mechanism.

To sum up, our results show for the first time that Ddr2 plays an essential role in the crosstalk between fat and bone. Molecular evidence indicated that Ddr2 regulates adipocyte lipolysis and bone mass via modulating Adcy5-cAMP-PKA pathway. Our observations unravel the potential of targeting adipocytic Ddr2 as a novel strategy for treating obesity and low-bone-mass disorders.

## Materials and methods

### Reagents and mice

Ascorbic acid (AA) and β-glycerol phosphate (β-GP) were obtained from sigma (St. Louis, MO, USA). Antibodies against phosphorylated Lipe (S853), total Lipe, and β-actin were obtained from Abcam (Cambridge, MA, USA. ab109400, ab45422, ab6276). SQ22536, AH7614 were obtained from Selleck (Shanghai, China). Adipoq-Cre mice were obtained from The Jackson Laboratory. *Ddr2* flox/WT mice (C57BL/6 background) were generously provided by Professor Gregory D Longmore in Washington University in St. Louis. To generate *Ddr2*^*Adipo*^ mice, *Ddr2* flox/flox mice were mated with Adipoq-Cre mice. Mice were housed in a facility with stable humidity and temperature and a 12 h:12 h light-dark cycle, with free access to water and food. Although no gender differences exist in phenotype, male mice were exclusively used. No randomization was used and all animal experimentation was approved by the Xi’an Jiaotong University Animal Care and Use Committee. All the analyses were done in a blinded fashion.

### Cell culture

Primary osteoblasts were extracted from 3–5-day-old neonatal calvariae with type two collagenase as previously described [47]. Calvaria cells were cultured in α-Modified Essential Medium (α-MEM) containing 10% fetal bovine serum (FBS). Osteoblast differentiation was induced with 50 μg/mL AA, and 10 mM β-GP. Mouse preadipocyte cell line 3T3-L1 was obtained from American Type Culture Collection (ATCC) and cultured in Dulbecco’s Modified Eagle’s Medium (DMEM), supplemented with 10% calf bovine serum (CBS).

### Co-culture of 3T3-L1 cells and calvaria cells

Preadipocyte 3T3-L1 cells were seeded in the transwell upper chamber, and induced with differentiation inducers for 6 d [48] followed by being treated with 10 μM isoproterenol for 3 h. Then the upper chamber was translocated to the plate where calvaria cells were cultured in advance.

### Bone marrow adipocyte progenitor cells isolation

Bone marrow cells were suspended with PBS containing 0.5% Bovine Serum Albumin (BSA) and 2 mM EDTA. Then an Adipose Tissue Progenitor Isolation kit (Miltenyi Biotec, San Diego, CA, USA) was used to isolate the adipocyte progenitor cells according to the manufacturer’s instruction. Briefly, cell suspension was incubated with Depletion Cocktail at 4 °C in the dark for 15 min. Then total reaction volume was adjusted to 500 μL, and cell suspension was applied onto a pretreated LS Column placed in the MACS Separator. Flow-through cells were collected and incubated with Isolation Cocktail at 4 °C in the dark for 15 min. Further positive selection was performed using a MS Column. After being fully washed, the positive cells were flushed out from the MS Column.

### Micro-computed tomography (μCT)

Mouse femurs were fixed in 10% neutral buffered formalin. μCT scanning was performed using a eXplore Locus SP system (GE Healthcare, Piscataway, NJ, USA). The trabecular bone samples ranging from just proximal to the distal growth plate to 20% of the bone length were analyzed. Bone parameters were calculated using Micview software (GE Healthcare), including bone mineral density (BMD), bone volume per tissue volume (BV/TV), trabecular number (Tb.No.), trabecular separation (Tb.Sp), trabecular thickness (Tb.Th.), cortical thickness (Ct.Th.), and cortical area (Ct. Ar.).

### Histology and histomorphometry

Epididymal WAT (white adipose tissue) was fixed in 10% formalin for 2 days and transferred to 70% ethanol. Sections were stained with hematoxylin and eosin (H&E) or Sirus Red. One representative section for each sample (mice) were scanned at 20× magnification. When analyzing the size of adipocytes using ImageJ (NIH), at least 2000 cells were counted per image, and 10 images were exported from one scanning for quantification. Cell-size distribution was analyzed in six samples (mice) for each group. Mouse femurs were fixed with 10% neutral buffered formalin, followed by decalcification in 14% EDTA for 2 weeks, then were paraffin embedded, sectioned, and stained by H&E, or tartrate-resistant acid phosphatase (TRAP). One representative section for each sample (mice) were scanned at ×4 and ×20 magnification. At least five region of interest (ROI) were analyzed per sample (mice). Static and dynamic histomorphometric parameters were measured by using a BioQuant OsteoII (BioQuant Image Analysis Corporation, Nashville, TN, USA). Osteoblast number and surface, as well as bone surface were measured for calculation of osteoblast number per bone surface (OB. No./BS) and osteoblast surface per bone surface (OB. S/BS). The number of individual osteoclasts and the percentage of osteoclast surface juxtaposed to bone were quantified as osteoclast number per bone surface (OC. No./BS) and osteoclast surface per bone surface (OC. S/BS). Number of bone marrow adipocyte and proportion were quantified by normalization to the marrow surface or area. All the analyses were done in a blinded fashion.

### Calcein labeling

8-week-old mice were injected intraperitoneally with 7.5 mg/kg calcein (Sigma, St. Louis, MO, USA) on days 6 and 2 before sacrifice. The fluorescence images of nondecalcified histological sections of femur were analyzed using BioQuant OsteoII (BioQuant Image Analysis Corporation, Nashville, TN). At least five region of interest (ROI) were analyzed per sample (mice). MAR (mineral apposition rate) and BFR/BS (bone formation rate/bone surface) were calculate as following: MAR = interlabel width/ day, BFR/BS = (double labeled surface + single labeled surface/2) × MAR/ (bone surface × 100). All the analyses were done in a blinded fashion.

### Glucose tolerance test (GTT) and insulin tolerance test (ITT)

GTT was performed by starving mice overnight with free access to water. On the next morning, mice were weighed and blood was obtained from lateral saphenous vein for quantification of glucose as baseline (time 0). Mice were then injected intraperitoneally with 50% sterile dextrose (1 mg/g bodyweight). Tail blood glucose was determined at 15, 30, 60, 90, and 120 min afterward using a Bayer Contour glucometer. For ITT, mice were starved with free access to water for 6 h. Then mice were weighed, and tail blood glucose were determined at 0, 15, 30, 45, 60, 90, and 120 min after intraperitoneally injected with human insulin at a dose of 0.5U/kg bodyweight.

### Body composition analysis

Body composition was measured using a Dual X-ray Digital System (Medicors, Korea) according to the manufacturer’s instructions. Body fat mass and lean mass were measured between 2 pm and 3 pm.

### Lipolysis assay

Twenty milligrams of epididymal visceral fat (vWAT) was collected and cultured in 200 μL of low-glucose Dulbecco’s Modified Eagle’s medium (DMEM) containing 2% fatty acid free BSA with or without 10 μM isoproterenol in a 96-well plate at 37 °C. One hour later, media were collected and glycerol release assay was performed according manufacturer’s instruction (Sigma, St. Louis, MO, USA). Tissue samples were harvested for protein quantification using BCA protein assay (Pierce, Rockford, IL, USA).

### Metabolites analysis

Bone marrow cells were extracted with isopropanol/hexane (2:3). The supernatant was dried completely in a vacuum concentrator without heating. Then methanol was added for sample derivatization. After nitrogen blow drying, hexane was added to dissolve the sample. Then the supernatant was analyzed by gas chromatograph system coupled with a mass spectrometer (GC-MS) using a GCMS-QP2010 Ultra (SHIMADAZU, Japan) which utilizes a CP-Sil88 capillary column. 1 μL aliquot of the analyte was injected in split mode (10:1). Helium was used as the carrier gas, with the front inlet purge flow as 5 mL min^−1^, and gas flow rate through the column as 1.05 mL min^−1^. The oven temperature ramp was as follows: 50 °C hold on 0 min, raised to 175 °C (20 °C /min) hold on 5 min, raised to 190 °C (5 °C/min) hold on 5 min, raised to 225 °C (5 °C/min) hold on 2 min, raised to 240 °C (5 °C/min) hold on 2 min. The front injection, transfer line, and ion source temperature were 240, 240, and 240 °C, respectively. The mass spectrometry data were acquired in scan mode after solvent delay of 4 min.

### Mitochondrial stress test

Mitochondrial respiration was measured and presented as the oxygen consumption rate (OCR) using a Seahorse XFe96 Extracellular Flux Analyzer (Agilent Technologies, Santa Clara, CA, USA). Calvaria cells were seeded in a Seahorse 96-well cell culture microplate at a density of 5 × 10^4^ cells/well, and induced with 50 mg/mL AA and 5 mM β-GP for 3 days. The cells were equilibrated to unbuffered DMEM (pH 7.4) containing 2.5 mM glucose, 50 mg/ml AA, and 5 mM β-GP at 37 °C for 1 h in a CO_2_-free incubator. The OCR measurement cycle consisted of 1.5 min mixing and 5 min measurement of the oxygen level. Three baseline OCR measurement cycles were followed by the sequential injection of oligomycin (1 mM), FCCP (2 mM), and a mixture of rotenone (1 mM) and antimycin A (1 mM). OCR was calculated by the Seahorse XFe96 software, Wave.

### RNA-seq library construction and sequencing

Total RNA was extracted using Trizol (Invitrogen, Carlsbad, CA, USA) according to the manufacturer’s protocol. RNA quality was assessed on an Agilent 2100 Bioanalyzer (Agilent Technologies, Palo Alto, CA, USA) and checked using RNase free agarose gel electrophoresis. After total RNA was extracted, eukaryotic mRNA was enriched by oligo (dT) beads, while prokaryotic mRNA was enriched by removing rRNA by Ribo-ZeroTM Magnetic Kit (Epicentre, Madison, WI, USA). Then the enriched mRNA was fragmented into short fragments using fragmentation buffer and reverse transcripted into cDNA with random primers. Second-strand cDNA were synthesized by DNA polymerase I, RNase H, dNTP and buffer. Then the cDNA fragments were purified with QiaQuick PCR extraction kit (Qiagen, Venlo, The Netherlands), end repaired, poly (A) added, and ligated to Illumina sequencing adapters. The ligation products were size selected by agarose gel electrophoresis, PCR amplified, and sequenced using Illumina HiSeq2500 by Gene Denovo Biotechnology Co. (Guangzhou, China).

### RNA-seq data acquisition, quality control, and processing

RNA-seq reads were filtered by fastp (version 0.18.0) to remove reads which contains adapters, >10% of unknown nucleotides, and >50% of low quality (Q-value ≤ 20) bases. After alignment with Ribosome RNA (rRNA) and Reference Genome using Short reads alignment tool Bowtie2 (version 2.2.8) and HISAT2. 2.4, respectively, the clean mapped reads of each sample were assembled using StringTie v1.3.1 in a reference-based approach, to quantify the gene abundance. For each transcription region, a FPKM (fragment per kilobase of transcript per million mapped reads) value was calculated to quantify its expression abundance and variations, using StringTie software. RNAs differential expression analysis was performed by DESeq2 software between two different groups (and by edgeR between two samples). The genes/transcripts with the parameter of false discovery rate (FDR) below 0.05 and absolute fold change ≥ 2 were considered differentially expressed genes/transcripts. Correlation analysis was performed by R. Correlation of two parallel experiments to evaluate the reliability of experimental results as well as operational stability. Principal component analysis (PCA) was performed with R package gmodels (http://www.rproject.org/). Gene Ontology (GO) enrichment analysis was performed using Gene Ontology database (http://www.geneontology.org/). Gene Set Enrichment Analysis (GESA) was performed using software GSEA and MSigDB to identify whether a set of genes in specific GO terms\pathways\DO terms shows significant differences in two groups. Briefly, we input gene expression matrix and rank genes by SinaltoNoise normalization method. Enrichment scores and *p-*value was calculated in default parameters.

### cAMP assay

Cells were lysed with cell lysis buffer. Supernatant was collected and assayed using a Colorimetric ELISA cAMP Assay Kit (Abcam, Cambridge, MA, USA). Briefly, cAMP standard or samples were added into the 96-well plate coated with anti-cAMP antibody. After a 10 min of incubation, 25 μL of HRP-cAMP conjugate working solution was added and incubated on shaker at room temperature for 3 h. Then the wells were fully washed, and Green Probe was added for incubation at dark for 3 h. Absorbance at 405 nm was recorded, and cAMP concentration was calculated according to the standard.

### PKA kinase activity assay

Cells were lysed with cell lysis buffer. Supernatant was collected and assayed using a PKA kinase activity Assay Kit (Abcam, Cambridge, MA, USA). Briefly, positive control or samples were added into the wells, followed by incubation with reconstituted ATP at 30 °C for 1 h. Then PKA phosphospecific substrate antibody was added and incubated at room temperature for 1 h. After being fully washed, IgG-HRP was added, and TMB substrate mediated detection was performed at 450 nm absorbance.

### EdU incorporation assay

Cell proliferation was assayed by 5-ethynyl-2′-deoxyuridine (EdU) incorporation kit (RiboBio, Guangzhou, China) according to the manufacturer’s instruction. Briefly, calvaria cells at 50% confluence were incubated with fresh medium containing 50 mM EdU for 2 h. Then cells were fixed with 4% formaldehyde and permeabilized with 0.5% Triton X-100 for 20 min at room temperature. After fully washing with PBS, 100 μL of Apollo® reaction cocktail was added for a 30 min of incubation. Then cells were stained with 4′,6-diamidino-2-phenylindole (DAPI) and visualized under a fluorescence microscope.

### RNA extraction and quantitative PCR (qPCR)

Total RNA was extracted using Trizol (Invitrogen, Carlsbad, CA, USA). Complementary DNA (cDNA) was synthesized from 2 μg of total RNA using the PrimeScript RT reagent (Takara, Japan). Quantitative qPCR was performed using the SYBR green PCR master mix (TaKaRa, Japan) and gene-specific primers. A 20 μL reaction mixture was prepared, containing 5 μL of cDNA, 10 μL of SYBR mix, and 200 nM concentrations of each primer. The 2^−ΔCT^ method was used for relative quantification of each target, and glyceraldehyde-3-phosphate dehydrogenase (GAPDH) was used as an endogenous control. The sequences of primers for various gene were shown in Supplementary Table 1.

### Western blotting

Cells were lysed in radioimmunoprecipitation assay (RIPA) buffer containing 20 mM Tris-HCl, pH 7.5, 150 mM NaCl, 1 mM EDTA, 1 mM EGTA, 1% Triton X-100, 2.5 mM sodium pyrophosphate, 1 mM β-glycerophosphate, 1 mM Na_3_VO_4_, 1 mM NaF, and protease inhibitor mixture (Roche Applied Science, Penzberg, Germany). Protein concentrations were measured using the BCA protein assay (Pierce, Rockford, IL, USA). The cell lysates were resolved by 10% sodium dodecylsulfate-polyacrylamide gel electrophoresis (SDS-PAGE) and transferred to PVDF membranes (Amersham, GE Healthcare, Little Chalfont, UK). After blocking in Tris-buffered saline containing 5% skim milk and 0.1% Tween-20, membranes were incubated with primary antibodies 4 °C overnight, followed by incubation with HRP-conjugated secondary antibodies. Enhanced chemiluminescence (ECL) detection solutions (Pierce, Rockford, IL, USA) were used to visualize the primary antibody-bound protein. Then the signals were detected by Bio-Rad ChemiDoc Imaging System.

### Statistical analysis

All the collected sample were analyzed using an unpaired two-tailed Student’s *t*-test in a blinded fashion. The results are representative of at least four independent experiments. Data are expressed as mean ± SEM. *P < 0.05, **P < 0.01, and ***P < 0.001 indicate the level of significance in all experiments.

## Supplementary information

Supplementary Material

## Data Availability

The datasets used and/or analyzed during the current study are available from the corresponding author on reasonable request.
