# Supplementary material for: Targeting adipocytic discoidin domain receptor 2 impedes fat gain while increasing bone mass
Source: Cell Death Differ. 2021 Oct 13;29(4):737–49. doi: 10.1038/s41418-021-00887-9 (PMC8990016; doi:10.1038/s41418-021-00887-9)
Supplement: Supplementary file 1 — Supplementary Material [file 41418_2021_887_MOESM1_ESM.pdf]

Fig. S1. Verification of Ddr2 knockout in adipocytes and other tissues. A Ddr2 mRNA levels. **B** Ddr2 protein levels. Data are presented as mean ± SEM. n=6/group. *** *P*<0.001 as determined by unpaired t test. BMA, bone marrow adipocyte; vis F, visceral fat; sub F, subcutaneous fat; BMMs, bone marrow macrophages.

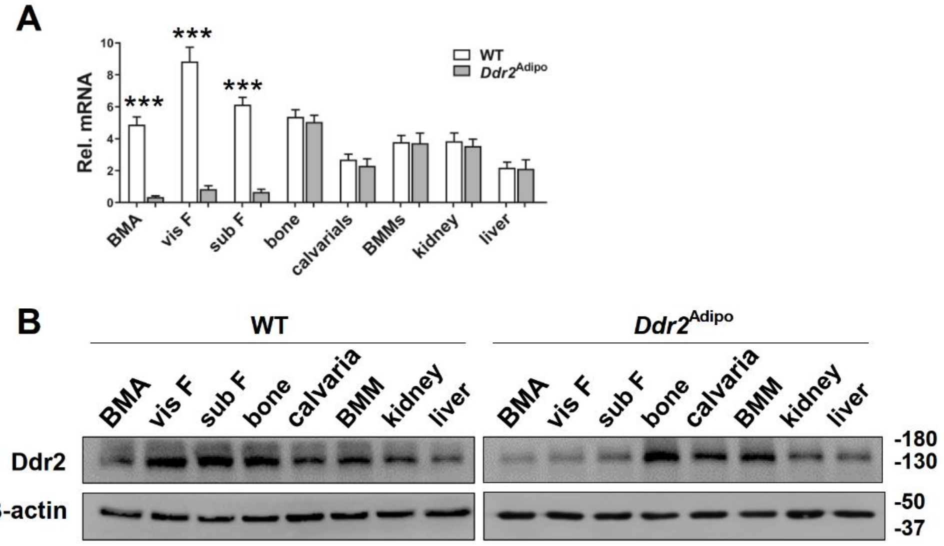

Fig. S2. Food intake of DDR2^{Adipo} and WT mice on HFD for 24 weeks. Data are presented as mean ± SEM. n=6/group.

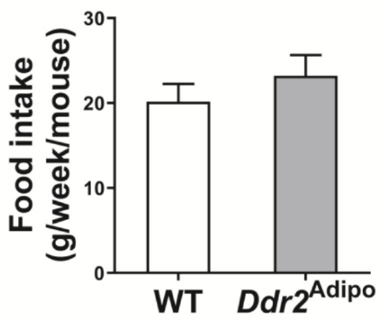

Fig. S3. Bone mass increase in DDR2^{Adipo} mice is not caused by the metabolic characteristics. **A** Serum adiponectin levels of DDR2^{Adipo} and WT mice. **B** Serum leptin levels. **C** Serum insulin levels. **D** Glucose tolerance test of 3-month old DDR2^{Adipo} and WT mice on chow diet. **E** Insulin tolerance test of 3-month old DDR2^{Adipo} and WT mice on chow diet. **F** Glucose tolerance test of DDR2^{Adipo} and WT mice on HFD. **G** Insulin tolerance test of DDR2^{Adipo} and WT mice on HFD. Data are presented as mean ± SEM. n=6/group. * P<0.05; ** P<0.01 as determined by unpaired t test.

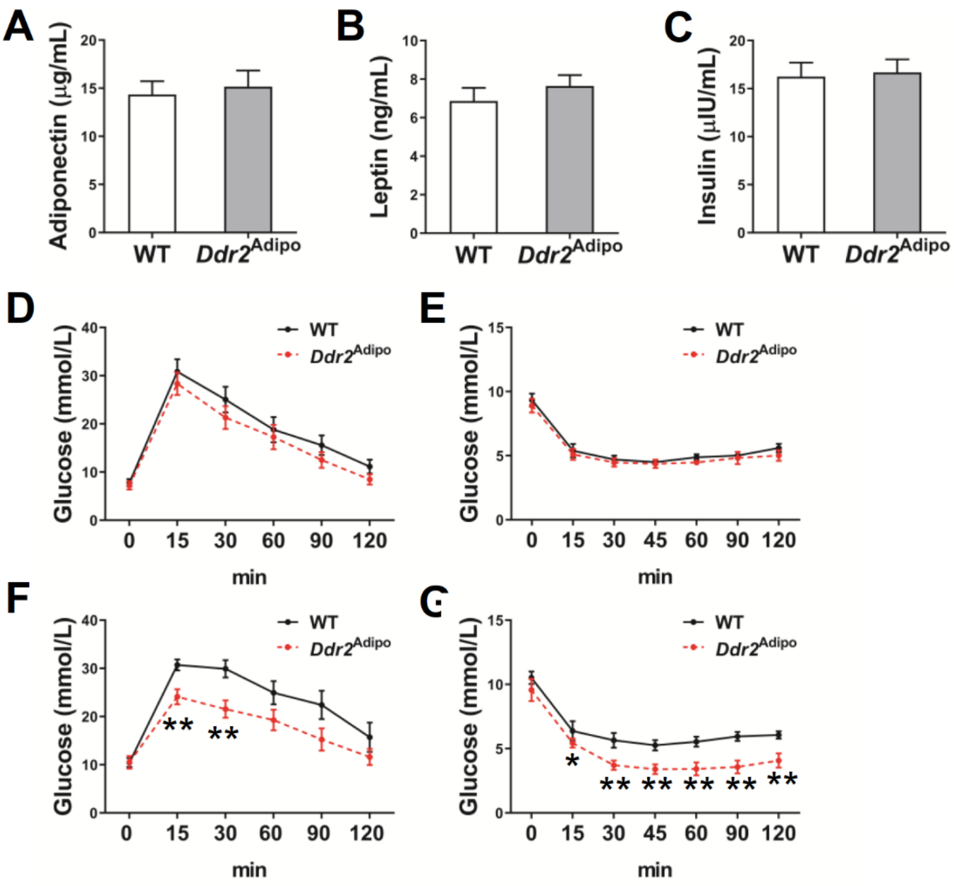

Fig. S4. No change in lineage differentiation of bone marrow mesenchymal cells. **A** mRNA expression of osteoblastic marker genes. **B** mRNA expression of adipocytic marker genes. Data are presented as mean ± SEM. n=6/group.

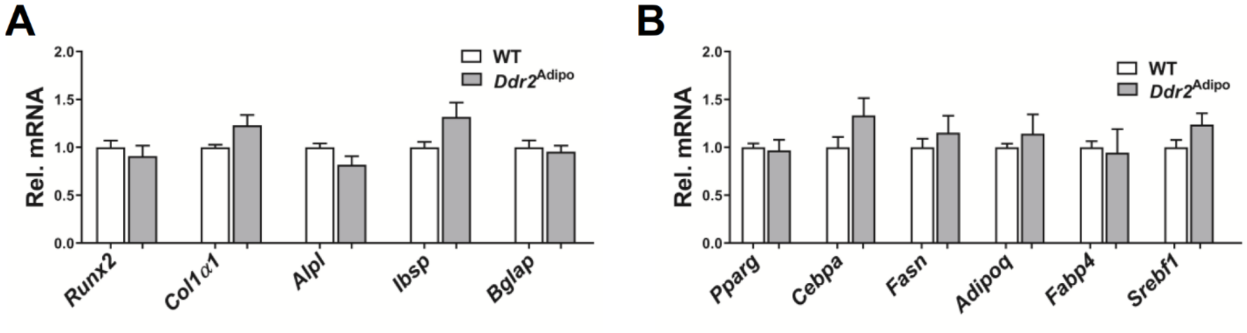

Fig. S5. Ddr2 knockdown in 3T3-L1 cells causes Adcy5 upregulation, cAMP-PKA-Lipe activation and consequent lipolysis. **A** mRNA level of *Adcy5* and *Ddr2*. **B** Protein level of *Adcy5* and *Ddr2*. **C** cAMP levels. **D** PKA activity. **E** Phosphorylated and total Lipe level. **F** FFA release. **G** Verification of *Adcy5* knockdown. **H** Verification of *Adcy5* overexpression. **I** Verification of *Ddr2* overexpression. Data are presented as mean ± SEM. n=6/group. * $P<0.05$; ** $P<0.01$; *** $P<0.001$ as determined by unpaired t test.

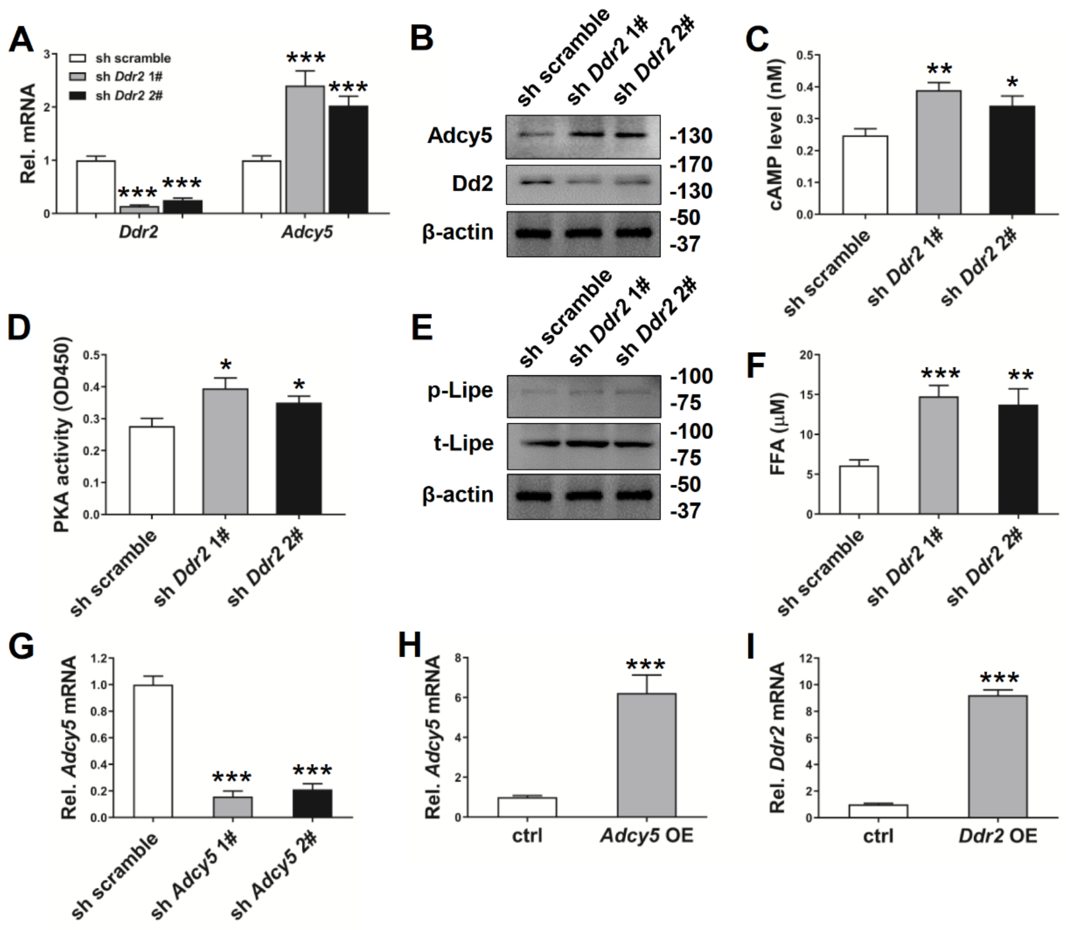

Supplementary Table 1 Primer list.

Gene	Forward primer	Reverse primer
Gapdh	TTCGACAGTCAGCCGCATCTTCTT	CAGGCGCCCAATACGACCAAATC
Runx2	CGCCCCTCCCTGAACTCT	TGCCTGCCTGGGATCTGTA
Col1a1	CGAAGGCAACAGTCGATTCA	CCCCAAGTTCCGGTGTGA
Alpl	ACACCAATGTAGCCAAGAATGTCA	GATTCGGGCAGCGGTTACT
Ibsp	CCGGCCACGCTACTTTCTT	GGACTGGAAACCGTTTC
Bglap	CTGACAAAGCCTTCATGTCCAA	GCGCCGGAGTCTGTTCACTA
Nfatc1	ATACCTGGCTCGGTAACACC	CATGCTCCAGTGCTGTCTTT
Itgb3	GATGACATCGAGCAGCTGAAAGAG	CCGGTCATGAATGGTGATGAGTAG
Acp5	ATGGGCGCTGACTTCATCAT	GGTCTCCTGGAACCTCTTGT
Dcst1	ACAAACAGTTCCAAAGCTTGC	TCCTTGGGTTCCTTGCTTC
Ctsk	CACTGGATAATTAAAAACAGCTGGG	CCAGGTTGGCAATGCCAC
Pparg	GGAGATCTCCAGTGATATCGACCA	ACGGCTTCTACGGATCGAAACT
Cebpa	TGGACAAGAACAGCAACGAC	TCACTGGTCAACTCCAGCAC
Fabp4	AAGAAGTGGGAGTGGGCTTT	ATGATCATGTTGGGCTTGGC
Srebf1	GATGTGCGAACTGGACACAG	CATAGGGGGCGTCAAACAG
Slc2a4	TAGGAGCTGGTGTGGTCAATACG	TAAAAGGGAAGGTGTCCGTCG
Fasn	CCACTGCTTACTACTCGTTA	AGGTATGCTCGCTTCTCT
Cd36	GGAACTGTGGGCTCATTGC	CATGAGAATGCCTCCAAACAC
Plin1	CTGTGTGCAATGCCTATGAGA	CTGGAGGGTATTGAAGAGCCG
Mogat1	TGGTGCCAGTTTGGTTCCAG	TGCTCTGAGGTCGGGTTCA
Gpam	CTTGGCCGATGTAAACACACC	CTTCCGGCTCATAAGGCTCTC
Dgat2	GCGCTACTTCCGAGACTACTT	GGGCCTTATGCCAGGAAACT
Acadl	TTTCCTCGGAGCATGACATTTT	GCCAGCTTTTTCCCAGACCT
Cpt1a	CCACCTCTTCTGCCTCTA	AACACCATAGCCGTCATC
Lipe	CTGAGATTGAGGTGCTGTC	GGTGAGATGGTAACTGTGAG
Pnpla2	GACGGAGAGAACGTCATCATATC	CCACAGTACACCGGGATAAAT
Mgll	TTCTGCTGACCGGCTTTG	GACGTGATAGGCACCTTCATAC
Lpl	CGCTCCATTCATCTCTTCA	CATCTTGCTGCTTCTCTTGebf

Prkg1	ATCCGAGAGGTCGAAGGATCT	ATTCCACGGGGTACATACAGT
Adcy5	CTTGGGGAGAAGCCGATTCC	ACCGCTTAGTGGAGGGTCT
Adcy6	TGAGTCTTCTAGCCAGCTCTG	CAGCACCAAGTAGGTGAACCC
Pla2g10	GTGCAGGTGTGACGAGGAG	CACTTGGGAGAGTCCTTCTCA
Adcy9	GCGTGAGGGTCAAGATCAACC	CATGGAGTCGAATTTGGGGTC
Agpat2	CAGCCAGGTTCTACGCCAAG	TGATGCTCATGTTATCCACGGT
Ptgs1	ATGAGTCGAAGGAGTCTCTCG	GCACGGATAGTAACAACAGGGA
Tshr	ACCCAGACTCTGAAGCTCAT	AGCTTCTGGTGTTCCGGATT
Plpp3	TCGTCCCTGAGAGTAAGAACG	TGCTTGTCTCGATGATGAGGAA
Slc27a1	CCGTATCCTCACGCATGTGT	CTCCATCGTGTCCTCAATGAC
Ffar1	CCTTCGCTCTCTATGTATCTGCC	CGCAGTTTAGCGTGGGACA
Ffar3	CTTCTTTCTTGGCAATTACTGGC	CCGAAATGGTCAGGTTTAGCAA
Ffar4	ACCAAGTCAATCGCACCCAC	CCAGCAGTGAGACGACAAAGA
Ddr2	CTGTCGGATGAGCAGGTTAT	CTCGGCTCCTTGCTGAAGAA
adipq	TACAACCAACAGAATCATTATGACGG	GAAAGCCAGTAAATAGAGTCGTTGA
